# Analysis of Ferroptosis-Mediated Modification Patterns and Tumor Immune Microenvironment Characterization in Uveal Melanoma

**DOI:** 10.3389/fcell.2021.685120

**Published:** 2021-07-27

**Authors:** Yi Jin, Zhanwang Wang, Dong He, Yuxing Zhu, Lian Gong, Mengqing Xiao, Xingyu Chen, Ke Cao

**Affiliations:** ^1^Department of Oncology, Third Xiangya Hospital of Central South University, Changsha, China; ^2^Department of Radiation Oncology, Hunan Cancer Hospital, The Affiliated Cancer Hospital of Xiangya School of Medicine, Central South University, Changsha, China; ^3^Department of Radiation Oncology, Hunan Key Laboratory of Translational Radiation Oncology, Hunan Cancer Hospital and The Affiliated Cancer Hospital of Xiangya School of Medicine, Central South University, Changsha, China; ^4^Department of Respiratory, The Second People’s Hospital of Hunan Province, Changsha, China

**Keywords:** Uveal melanoma, ferroptosis, tumor-infiltrating immune cells, tumor immune environment, prognostic model

## Abstract

Uveal melanoma (UVM) is an intraocular malignancy in adults in which approximately 50% of patients develop metastatic disease and have a poor prognosis. The need for immunotherapies has rapidly emerged, and recent research has yielded impressive results. Emerging evidence has implicated ferroptosis as a novel type of cell death that may mediate tumor-infiltrating immune cells to influence anticancer immunity. In this study, we first selected 11 ferroptosis regulators in UVM samples from the training set (TCGA and GSE84976 databases) by Cox analysis. We then divided these molecules into modules A and B based on the STRING database and used consensus clustering analysis to classify genes in both modules. According to the Gene Ontology (GO), Kyoto Encyclopedia of Genes and Genomes (KEGG), and Gene Set Enrichment Analysis (GSEA), the results revealed that the clusters in module A were remarkably related to immune-related pathways. Next, we applied the ESTIMATE and CIBERSORT algorithms and found that these ferroptosis-related patterns may affect a proportion of TME infiltrating cells, thereby mediating the tumor immune environment. Additionally, to further develop the prognostic signatures based on the immune landscape, we established a six-gene-regulator prognostic model in the training set and successfully verified it in the validation set (GSE44295 and GSE27831). Subsequently, we identified the key molecules, including ABCC1, CHAC1, and GSS, which were associated with poor overall survival, progression-free survival, disease-specific survival, and progression-free interval. We constructed a competing endogenous RNA network to further elucidate the mechanisms, which consisted of 29 lncRNAs, 12 miRNAs, and 25 ferroptosis-related mRNAs. Our findings indicate that the ferroptosis-related genes may be suitable potential biomarkers to provide novel insights into UVM prognosis and decipher the underlying mechanisms in tumor microenvironment characterization.

## Introduction

Uveal melanoma (UVM), which represents 85% of ocular melanomas, is the most common primary intraocular malignancy in adults and arises from melanocytes of the iris (3–5%), ciliary body (5–8%), or choroid (∼85%) (Chang et al., 1998; McLaughlin et al., 2005). Analysis of the Surveillance, Epidemiology, and End Results program database in the USA and the European Cancer Registry-based study on survival and care of cancer patients revealed an overall incidence of 1.3–8.6 cases per million per year (Virgili et al., 2007; Singh et al., 2011). Currently, the management of surgical options mainly depends on tumor location, extent, and size, adverse effects, and systemic status, including partial iridectomy, iridotrabeculectomy of the iris, and iridocyclectomy (Henderson and Margo, 2008). Radiotherapy can also achieve high local control in most cases with extensive seeding and in non-resectable tumors (Shields et al., 2013; Rahmi et al., 2014). However, approximately 50% of patients develop metastatic disease, predominantly in the liver, which is refractory to traditional treatments such as chemotherapy and surgical metastasectomy (Achberger et al., 2014). Due to the lack of standard therapies to significantly improve survival, newer fields of targeted treatments and immunotherapies have rapidly emerged and yielded impressive results by targeting specific regulators or immune system checkpoints (Chang et al., 1998; de Vries et al., 1998; Chen et al., 2017). However, there are few reports on oncogenes underlying UVM. Therefore, unraveling genomic properties is crucial for the development of effective treatments and prediction of individual survival.

Previous studies have confirmed that programmed cell death (PCD) is related to tumorigenesis, progression, and metastasis processes (Labi and Erlacher, 2015; Lee et al., 2018). Efficient cancer treatment ultimately aims to induce cell-specific PCD. Ferroptosis is a novel form of PCD that is driven by lethal iron-dependent accumulation of lipid reactive oxygen species (ROS). Cytological changes, including decreased mitochondrial cristae, a ruptured outer mitochondrial membrane, or a condensed mitochondrial membrane, ferroptosis gradually plays a pivotal role in the mediation of the carcinogenic environment and suppression of tumor progression in various cancer cells (Yagoda et al., 2007; Cao and Dixon, 2016; Latunde-Dada, 2017; Yu et al., 2017). For instance, results from NCI-60, a panel of different cancer cell lines from eight different tissue types, showed that ferroptosis with excess iron overload was found in several types of tumor cells such as breast cancer, ovarian cancer, lung cancer, diffuse large B-cell lymphoma, and renal cell carcinoma (Xie et al., 2016). Moreover, further investigations revealed that radiotherapy and chemotherapeutic drugs such as cisplatin and temozolomide, combined with ferroptosis-induction therapy, had a more significant effect than traditional treatments alone (Mou et al., 2019). Currently, no studies have focused on the role of ferroptosis in UVM patients and rare therapies inducing ferroptosis-induction therapy; thus, we first performed a set of studies to identify the clinical importance of ferroptosis in UVM and estimate the relationship between the microenvironment and specific ferroptosis-related biomarkers, aiming to explore the essential function of ferroptosis.

Checkpoint inhibitors, which promote immune function and induce anti-tumor immunity, are among the most successful agents for treating advanced melanoma. Regarding UVM, statistics from a phase II trial of metastasized patients showed a satisfactory outcome with a disease control rate (DCR) of 47% and a durable DCR of 21% ([DCR] = complete response [CR] + partial response [PR] + stable disease [SD], durable DCR [DCR ≥ 6 months]) (Schank and Hassel, 2019; Pelster et al., 2021; Piulats et al., 2021). Despite sharing similar clinical characteristics, patients with UVM may invariably exhibit different clinical survival rates (Polak et al., 2007). The underlying molecular mechanisms that elucidate this phenomenon may be attributed to the tumor microenvironment (TME), which includes cancer cells, stromal cells, distant recruited cells, bone marrow-derived cells, and secreted factors (Topalian et al., 2012; Wang et al., 2017). Thus, identifying TME characteristics is crucial to improving clinical survival outcomes in patients with UVM. Emerging evidence suggests that ferroptosis may be an adaptive process for achieving better treatment effects, such as exerting an obvious synergistic effect with PD1/PD-L1/CTLA4 inhibitors by generating lipid-derived mediators to modulate intracellular and intercellular signaling pathways, including receptor tyrosine kinase signaling in the TME (Friedmann et al., 2019; Hoefsmit et al., 2020). Moreover, some efficient treatments, including radiotherapy and immunotherapy, control ferroptosis and promote immunotherapy-activated CD8+ T cells, and radiotherapy-activated ATM can effectively kill cancer cells and prevent cancer cells from escaping immune surveillance (Lang et al., 2019). However, little is known about the relationship between ferroptosis in the tumor immune microenvironment and UVM.

In this study, we performed a retrospective analysis based on The Cancer Genome Atlas (TCGA) database and the Gene Expression Omnibus Database (GEO) to estimate the effect of ferroptosis-related genes on the prognostic value. We further verified its prognostic significance and explored the underlying mechanisms between ferroptosis-related genes and individual TME characterizations.

## Materials and Methods

### Data Processing of UVM Dataset

We downloaded the RNA sequencing, mutation expression, and clinical information of UVM from TCGA and GEO databases. The RNA sequencing data (FPKM value) and somatic mutation data from TCGA-UVM were downloaded from the Genomic Data Commons^1^. In total, three eligible GEO datasets (GSE27831, GSE44295, GSE84976) were downloaded and received background adjustment and quantile normalization using affy and simpleaffy packages. Data from TCGA and GEO are publicly available.

### Selection of Potential Ferroptosis-Related Genes

We first selected 60 ferroptosis-related genes (Supplementary Table 1) from previously published articles (Stockwell et al., 2017; Bersuker et al., 2019; Doll et al., 2019; Hassannia et al., 2019). Cox proportional hazard regression analysis was used to select the prognostic regulators positively or negatively based on the TCGA dataset and GSE84976. Finally, we considered the intersection of these regulators from different databases as potential functional molecules.

### Clustering of Ferroptosis-Related Regulators

The STRING database^2^ was used to analyze protein–protein interactions (PPI) among ferroptosis modulators. In this study, we propose an MCL cluster to identify cancer driver modules that combine mutex, functional similarity, and connectivity in the PPI network (Zhang et al., 2020). Pearson correlation analysis was used to reveal the associations among different regulators in different modules. Next, differentially expressed prognostic ferroptosis-related genes in specific modules were classified into different clusters using the ConsensusClusterPlus R package with optimal k-means clustering (Wilkerson and Hayes, 2010), which aimed to precisely gather genes with similar expression, and we performed 1,000 iterations (50 iterations, resampling rate of 80%) to ensure the stability of the classification. Overall survival (OS) analysis between different clusters was calculated using the Kaplan–Meier method.

### Identification of Signaling Pathways in Ferroptosis-Related Patterns

To identify the function regulated by diverse patterns, we obtained the differentially expressed genes (DEGs) with | logFC (fold change)|≥2 and adj. *p* < 0.05 by applying the empirical Bayesian approach of limma R package in the standard comparison mode. To further investigate the signaling pathways in ferroptosis-related patterns, we implemented the clusterProfiler R package to perform the Kyoto Encyclopedia of Genes and Genomes (KEGG) pathway, Gene Ontology (GO) biological processes, and Gene Set Enrichment Analysis (GSEA) analysis, which aimed to evaluate all DEGs and assess the functions associated with subtypes.

### Estimation of Immune Cell Infiltration in Ferroptosis-Related Patterns

In this study, we applied the ESTIMATE algorithm. The R script was downloaded from the website^3^ and applied to estimate the ratio of the immune stromal components in the TME and further explore differences in the TME scores, including ESTIMATE, immune, and stromal scores (Newman et al., 2015). We utilized the CIBERSORT package to assess the distribution of 22 immune cell types based on TCGA-UVM samples. Results with *p* < 0.05 obtained from the ESTIMATE algorithm and CIBERSORT analysis were used in further analysis.

### Construction of Prognostic Ferroptosis Signatures and Validation

Univariate Cox regression analysis was performed to examine the relationship between ferroptosis-related genes and OS in the TCGA UVM cohort. Moreover, two prognostic models were constructed, and the risk score was generated as follows: risk score = Expression_mRNA1_ × Coefficient_mRNA1_ + Expression_mRNA2_ × Coefficient_mRNA2_ +…Expression_mRNAn_× Coefficient_mRNAn_. The patients from TCGA and GEO databases were divided into high-risk and low-risk groups according to the median cutoff of the risk score. Next, to determine independent prognostic factors in UVM, univariate and multivariate Cox analyses were utilized to distinguish the clinicopathological parameters positively or negatively with the hazard ratio (HR). Subsequently, the Kaplan–Meier survival method was used to screen the availability of the prognostic model, and the receiver operating characteristic (ROC) curve was used to evaluate the prediction accuracy of 3-year and 5-year OS. To enhance prediction accuracy and interpretability, three GEO cohorts were further presented as validation sets to re-verify the prognostic model and selection of key ferroptosis modulators.

### Building of ceRNA Network Based on Key Genes

To further understand how the ferroptosis-related mRNAs regulate tumorigenesis along with miRNA and lncRNA, we obtained differentially expressed lncRNAs and miRNAs based on the TCGA cohort with the standards of | log2(Fold change)|>1 and *p* < 0.05 by using the R package “limma.” Furthermore, we used the miRcode database to target experimentally validated lncRNA (Jeggari et al., 2012) and miRTarBase^4^, miRDB^5^, and TargetScan^6^ datasets (Li et al., 2014; Agarwal et al., 2015; Chou et al., 2018; Chen and Wang, 2020) to predict miRNA–mRNA interactions. The competing endogenous RNA (ceRNA) network was visualized using the Cytoscape software (Shannon et al., 2003).

### Statistical Analysis

Most analyses were performed using the R software (version 3.6.1^7^). Kaplan–Meier curves were used to compare OS based on the risk score of subgroups and expression of ferroptosis-related genes. The comparison of the ESTIMATE algorithm between the two subgroups was performed using the Wilcoxon rank sum test. Cox proportional hazard regression analyses were used to select the independent prognostic value of OS, including clinical characteristics and ferroptosis regulators. All statistical *p* values were bilateral, and *p* < 0.05 was considered statistically significant.

## Results

### Selection of Prognostic Ferroptosis-Related Regulators

**FIGURE 1:**
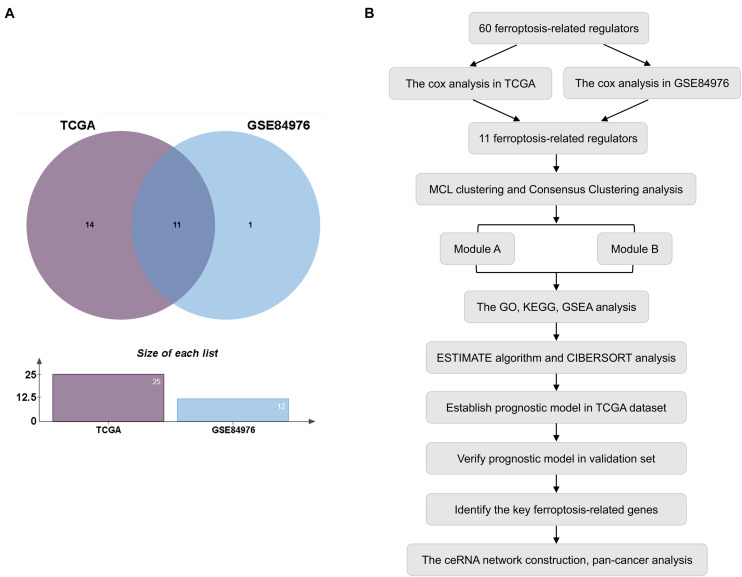
Selection of prognostic regulators. **(A)** Venn plot of ferroptosis-related regulators. **(B)** Study flow chart.

In this study, the expression of 60 ferroptosis-related regulators and clinicopathological characteristics associated with UM patients were obtained from the TCGA and GEO databases. First, 80 UM patients from TCGA and 28 UM patients from GSE84976 with OS were identified as the training set for selecting potential ferroptosis-related modulators. We then utilized Cox analysis to identify these genes and found 11 genes, including 9 upregulated genes (CHAC1, NQO1, SQLE, SLC1A5, GSS, LPCAT3, GPX4, AIFM2, and ABCC1) and 2 downregulated genes (ACSF2 and FDFT1) that were overlapped in TCGA and GSE84976 datasets (Figure 1A and Supplementary Table 2). The workflow is illustrated in Figure 1B.

### Clustering of Ferroptosis-Related Genes With Prognostic Value of Clinical Outcomes

**FIGURE 2:**
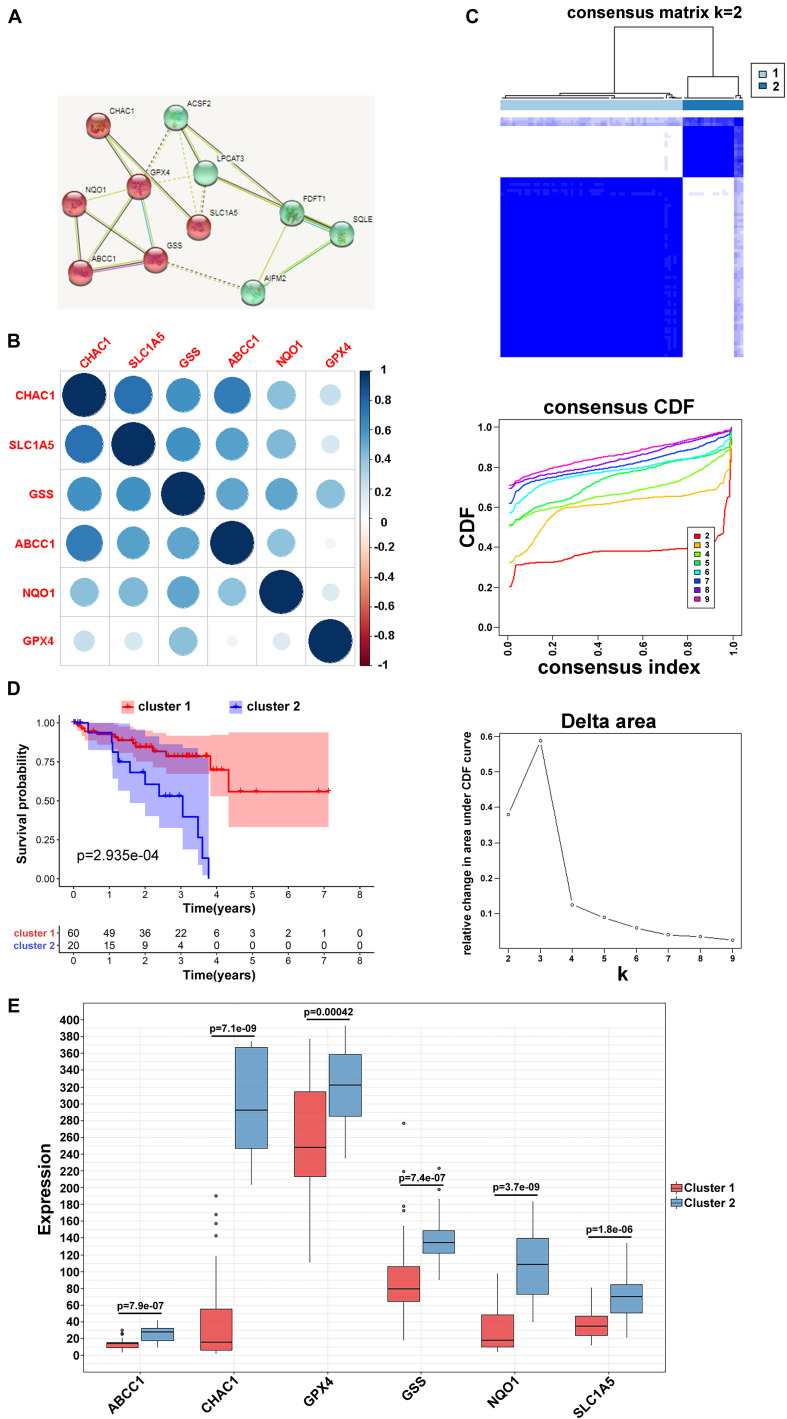
Clustering of ferroptosis-related genes. **(A)** MCL clustering of ferroptosis-related genes. **(B)** Pearson correlation analysis among ferroptosis-related regulators in module A. **(C)** Consensus clustering cumulative distribution function (CDF) and relative change in area under CDF curve for *k* = 2 in module A. **(D)** Kaplan–Meier curves of two clusters in UVM about OS in module A. **(E)** The different expression of the ferroptosis-related mediators in two clusters based on module A.

Based on the STRING database, differentially expressed or prognostic ferroptosis-related genes were selected for MCL clustering and divided into cancer driver modules A and B (Figure 2A). Pearson correlation analysis showed that genes in both functional modules were highly correlated (Figure 2B and Supplementary Figure 1A). To better understand the interrelations among these regulators, a consensus clustering analysis using the Consensus Cluster Plus package was implemented in the TCGA UVM cohort, and *k* = 2 seemed to be the most appropriate selection in both modules (Figure 2C and Supplementary Figure 1B). Next, the Kaplan–Meier method showed a significant difference in OS when cluster1 with low risk was compared to cluster2 with high risk (Figure 2D and Supplementary Figure 1C). Moreover, to clearly visualize the expression of these regulators, we plotted a boxplot based on module A and found that the expression of CHAC1, ABCC1, NQO1, GPX4, SLC1A5, and GSS in cluster2 was higher than that in cluster1 (*p* < 0.01) (Figure 2E), while in module B, the expression of FDFT1 and ACSF2 was higher in cluster2 than in the other clusters (Supplementary Figure 1D).

### Interaction of Ferroptosis-Related Regulators in Two Patterns

**FIGURE 3:**
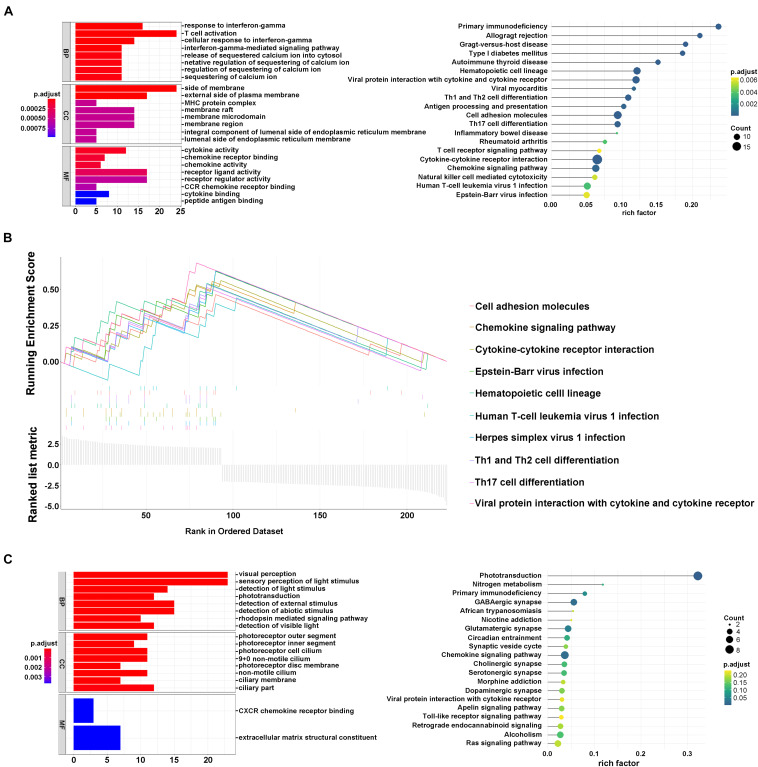
Interaction of ferroptosis-related genes. **(A)** GO and KEGG analyses of DEGs in module A. **(B)** GSEA analysis of DEGs in module A. **(C)** GO and KEGG analysis of DEGs in module B.

To investigate the potential biological processes involved in the molecular heterogeneity between the clusters, we identified DEGs with | log2 (fold change) |>2 and adj. *p* < 0.05, in the TCGA cohort. In module A, the biological process of GO analysis showed that the DEGs were enriched in response to interferon-gamma, T-cell activation, and cellular response to interferon-gamma. Cellular component analysis indicated that DEGs were abundant on the side of the membrane and on the external side of the plasma membrane. The molecular function indicated that DEGs were mainly located in receptor regulator activity and ligand activity. KEGG analysis showed that the DEGs were enriched in primary immunodeficiency, Th1, Th2, Th17 cell differentiation, and so on (Figure 3A). Furthermore, according to the results from GSEA analysis, these DEGs were primarily enriched in these terms, which were significantly related to cluster1: cell adhesion molecules, Th1 and Th2 cell differentiation, and Th17 cell differentiation. These results have been confirmed to be remarkably related to immunotherapy, giving us some insights into the ferroptosis-regulating effects on the tumor immune microenvironment (Figure 3B; Im et al., 2016; Garcia-Diaz et al., 2017; Zhang et al., 2017). However, the GO, KEGG, and GSEA analyses in module B failed to target meaningful pathways associated with the immune landscape (Figure 3C).

### Characteristics of Tumor Immune Microenvironment in UVM Patients

**FIGURE 4:**
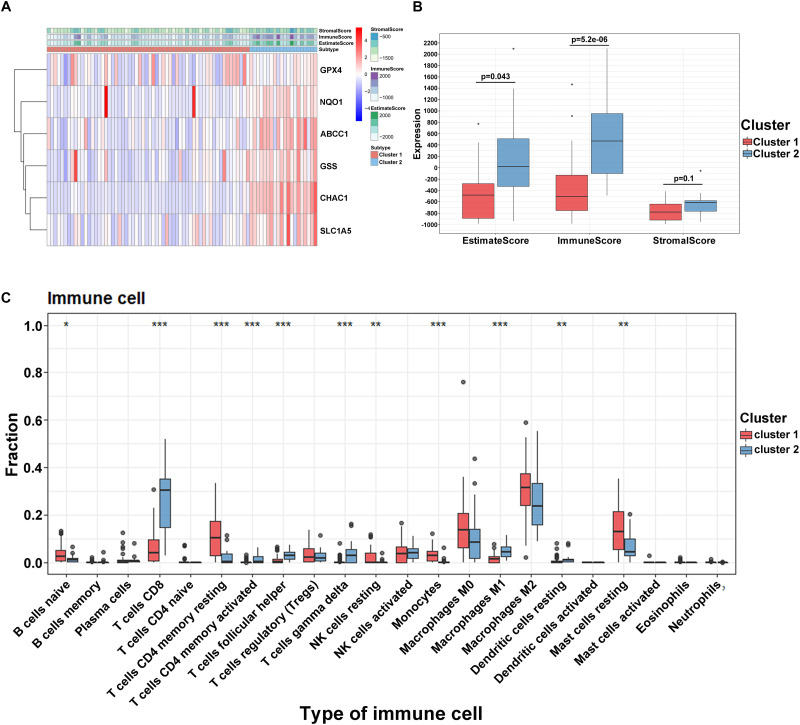
Tumor immune microenvironment in module A. **(A)** The heatmap of ferroptosis-related regulators in two clusters. **(B)** The different expression of ESTIMATE score in two clusters. **(C)** The different distribution of 22 TME infiltrating cells in two patterns (***p < 0.001; **p < 0.01; *p < 0.05).

To reveal the potential role and explore the potential mechanism of ferroptosis modification-related phenotypes in the TME, we used the ESTIMATE algorithm to apply stromal scores, immune scores, and ESTIMATE scores for all UVM samples. As shown in Figure 4A, we found that all enrolled genes in module A showed a similar expression trend along with immune and ESTIMATE scores. Moreover, in this study, when compared to cluster1, the ESTIMATE (*p* = 0.043) and immune scores (*p* = 0.52e^–6^) were significantly higher in cluster2, indicating that ferroptosis-related patterns may be involved in regulating the immune landscape and play a central role in immune regulation (Figure 4B). However, the mechanism by which ferroptosis-related patterns affect immune-related molecules (especially TMEs) is unclear. Therefore, we quantified the level of immune cell infiltration to evaluate the immune landscape of clusters using the CIBERSORT algorithm. The results revealed that naïve B cells, CD8 T cells, CD4 memory resting T cells, CD4 memory activated T cells, follicular helper T cells, and gamma delta T cells accounted for a large proportion of immune cell infiltration. Particularly, we observed that cluster1 with better survival showed higher levels of naïve B cells, resting T cells CD4 memory, resting NK cells, resting monocytes, and resting mast cells compared to cluster2. On the contrary, the outcome revealed that the proportion of CD8 + T cells, CD4 memory activated T cells, follicular helper T cells, gamma delta T cells, macrophages M0, and resting dendritic cells were higher in cluster2 (Figure 4C). However, we failed to unravel the relationship between module B and the immune microenvironment (Supplementary Figure 2B). These results suggest that specific multiple ferroptosis-related modulators may combine to affect the type of TME infiltrating cells to mediate the TME and change the clinical survival in UVM.

### Prognostic Analysis of Risk Model and Ferroptosis-Related Genes

**FIGURE 5:**
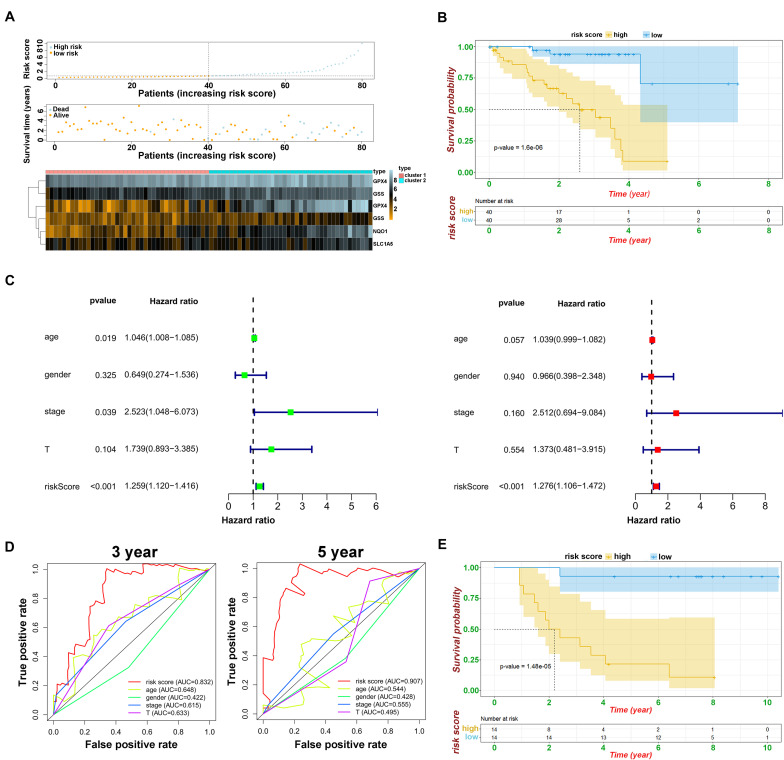
Prognostic analysis of risk model and ferroptosis-related genes. **(A)** The distributions of risk scores, alive/dead status, and expression of ferroptosis-related genes in module A. **(B)** Kaplan–Meier curves of patients in high/low risk about OS in module A. **(C)** Univariate (above) and multivariate analysis (below) of clinical characteristics. **(D)** ROC curve of risk score and clinical characteristics in module A. **(E)** Kaplan–Meier curves of patients in high/low risk about OS based on GSE84976.

To develop a predictive signature based on the immune landscape of UVM patients, we performed Cox regression analysis on six modulators in module A using the TCGA database. Next, we calculated the risk score as follows: risk score = CHAC1 × 0.0037 + ABCC1 × 0.067 + NQO1 × 0.0067 + GPX4 × 0.0043 + SLC1A5 × 0.021 + GSS × 0.012. The risk score distribution, survival status, and expression profile of the six prognostic regulators are shown in Figure 5A. Patients were separated into the low-risk or high-risk groups with the median cutoff, and we found that the high-risk group had a significantly poorer OS (*p* = 1.6e^–08^) (Figure 5B). Univariate and multivariate analyses showed that clinical characteristics, including age, sex, and T stage, were unable to predict OS, except for the risk score, which was viewed as an independent prognostic factor (*p* < 0.001, HR = 1.276, 95% CI = 1.106–1.472) (Figure 5C). The results from the ROC curve indicated that the risk score has strong predictive ability, with an AUC of 0.832 and 0.907 at 3 and 5 years, respectively (Figure 5D). These results indicate that the ferroptosis-related risk model from module A may serve as an important indicator for evaluating the prognosis of UVM. Additionally, in module B, we also constructed the risk score as follows: risk score = FDFT1 × (−0.012) + SQLE × 0.016 + AIFM2 × 0.05 + LPCAT3 × 0.17 + ACSF2 × (−0.19). As shown in Supplementary Figures 3A,B, we found that as the risk score increased, more patients died. The ROC curve showed that the risk score risk score had a better efficiency in predicting 3- and 5-year OS, with AUC values of 0.828 and 0.792, respectively (Supplementary Figure 3C). Furthermore, in one of the training sets GSE84976, we also obtained satisfactory outcomes, and both ferroptosis-related models could be regarded as independent risk factors for predicting OS (Figure 5E and Supplementary Figure 3D).

### Verification of Ferroptosis Regulators–Based Risk Model

**FIGURE 6:**
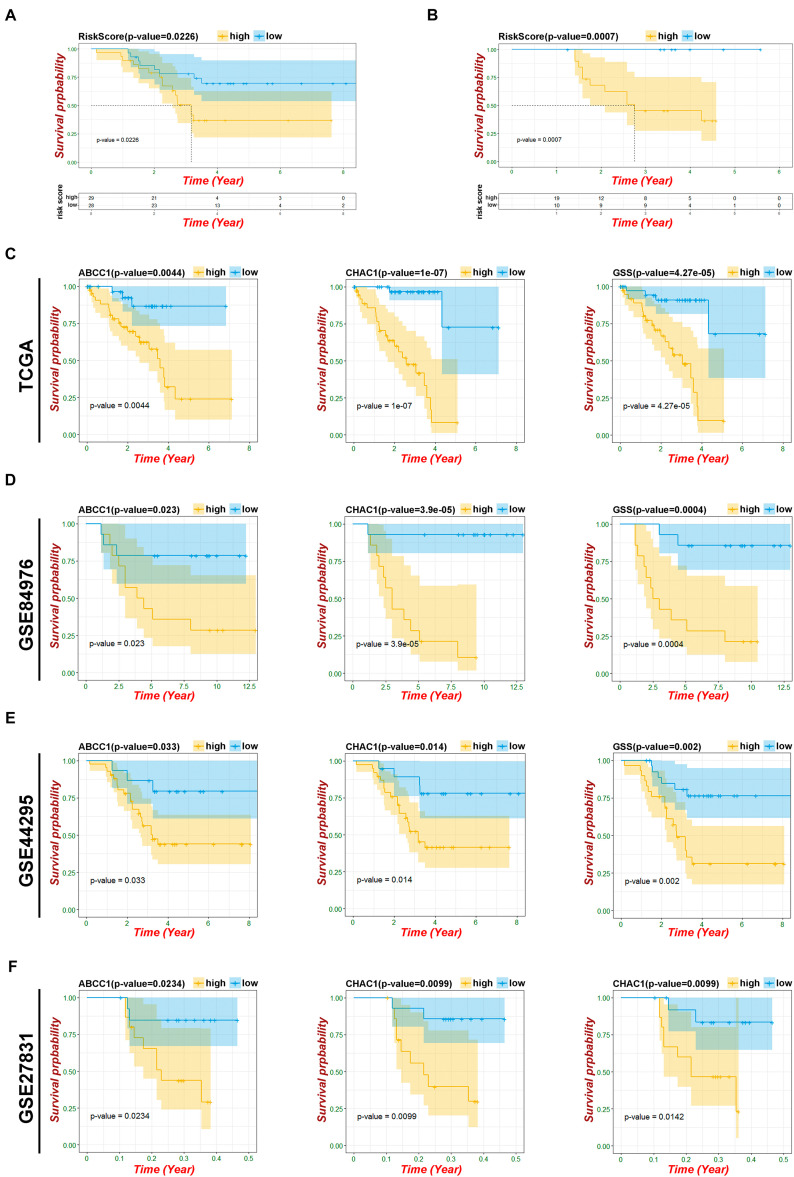
Verification of risk model and ferroptosis-related genes. **(A)** Kaplan–Meier curves of patients in risk model about OS based on GSE44295. **(B)** Kaplan–Meier curves of patients in risk model about PFS based on GSE27831. **(C)** Kaplan–Meier OS curves for patients in key ferroptosis-related genes based on TCGA. **(D)** Kaplan–Meier OS curves for patients in key ferroptosis-related genes based on GSE84976. **(E)** Kaplan–Meier OS curves for patients in key ferroptosis-related genes based on GSE44295. **(F)** Kaplan–Meier PFS curves for patients in key ferroptosis-related genes based on GSE27831.

To verify the predictive ability of both risk models, validation analysis was performed in two GEO datasets, including 57 UM from GSE44295 with OS and 29 patients from GSE27831 with progression-free survival (PFS). In GSE44295, which contained the largest sample of UVM in the Genomic Data Commons, the Kaplan–Meier curve revealed that there was a significant difference between the high-risk and low-risk groups with a log-rank test of *p* = 0.026 (Figure 6A). The risk formula from module A was exactly suitable for predicting OS by using the median value as the cutoff. Similarly, in GSE27831, the Kaplan–Meier analysis indicated that this risk formula can be predictive of a high risk of disease progression with a log-rank test of *p* = 0.0007 by using the best cutoff (Figure 6B). However, the risk formula from module B failed to be re-verified in both GEO databases. To further select the key prognostic ferroptosis modification-related genes, we continued to calculate the prognostic values of six genes in all GEO datasets and believed that the overlapping molecules might be significantly meaningful. As shown in Figures 6C–F, ABCC1, CHAC1, and GSS were successfully re-verified as crucial biomarkers to induce poor OS and PFS in TCGA and all GEO datasets. Additionally, data from the UCSC Cancer Genomics Browser^8^ showed that high ABCC1, CHAC1, and GSS expression was associated with decreased disease-specific survival (DSS) and progression-free interval (PFI) (*p* < 0.001) (Supplementary Figure 4).

### Construction of ceRNA Based on Key Genes

**FIGURE 7:**
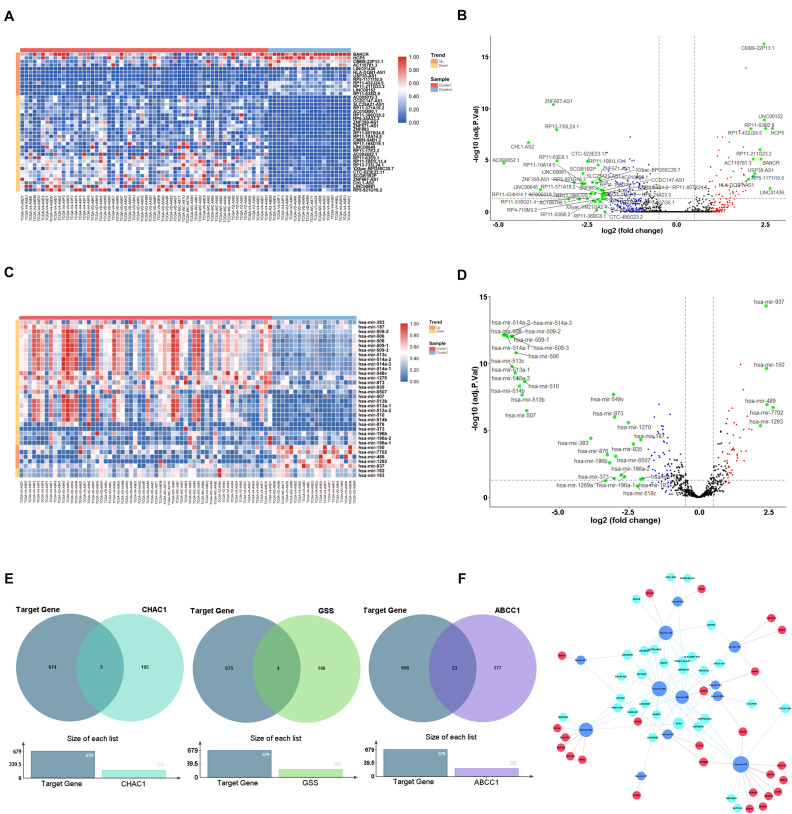
Construction of ceRNA network key genes. **(A)** Heatmap of differential expression lncRNAs. **(B)** Volcano plot of differential expression lncRNAs. **(C)** Heatmap of differential expression miRNAs. **(D)** Volcano plot of differential expression miRNAs. **(E)** Venn plot of target mRNAs and highly associated ferroptosis-related regulators. **(F)** ceRNA building of the 29 lncRNAs (light blue) and 12 miRNAs (dark blue) and 25 mRNAs (red).

To elucidate the mechanism underlying the ferroptosis-related pathway combined with miRNAs and lncRNAs, we obtained 316 differentially expressed lncRNAs and 126 miRNAs using the TCGA database (Supplementary Tables 3, 4). We drew the heatmaps (Figures 7A,C) and volcano plots (Figures 7B,D) to visualize the distribution of DEGs with | logFC (fold change) |≥2 and FDR < 0.05. To further identify the pathway by which lncRNAs mediate mRNA expression by sponging miRNAs, we selected 29 lncRNAs from the miRcode database, which targeted 20 miRNAs and enrolled a total of 679 mRNAs based on three databases (miRTarBase, miRDB, and TargetScan) (Supplementary Table 5). We then calculated the top 200 genes that were highly associated with ABCC1, CHAC1, and GSS to take the intersection of the enrolled mRNAs (Figure 7E); finally, we constructed a ceRNA regulatory network containing 25 ferroptosis-related mRNAs, 12 miRNAs, and 29 lncRNAs and then used Cytoscape for visualization (Figure 7F).

### Evaluation of Key Genes in Immune Landscape and Pan-Cancer

**FIGURE 8:**
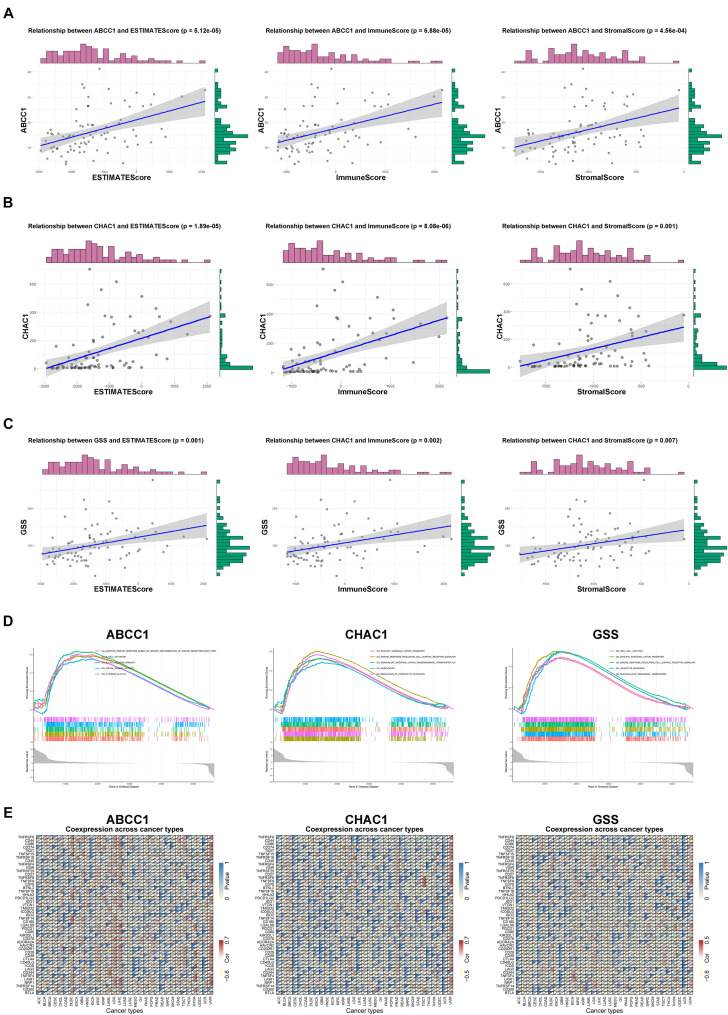
Evaluation of key genes in immune landscape. **(A)** Relationship between ABCC1 expression and ESTIMATEScore, ImmuneScore, as well as StromalScore. **(B)** Relationship between CHAC1 expression and ESTIMATEScore, ImmuneScore, as well as StromalScore. **(C)** Relationship between GSS expression and ESTIMATEScore, ImmuneScore, as well as StromalScore. **(D)** GSEA analysis of ABCC1, CHAC1, and GSS in UVM. **(E)** Correlation among ABCC1, CHAC1, and GSS expression and immune-related mRNAs.

To further evaluate the role of key genes in mediating the immune signature, we analyzed the correlation between the expression of ABCC1, CHAC1, GSS, and ESTIMATE scores and infiltrating immune cells. The results showed that ABCC1, CHAC1, and GSS levels had a significantly positive correlation with immune score, stromal score, and ESTIMATE score (Figures 8A–C). In addition, we found that the proportion of diverse types of T cell and B cell naïve dendritic cells, which have been proven to coordinately trigger successful immune reactions and positive costimulatory signals, had an intimate relationship with the expression of three key genes (Sharpe and Pauken, 2018; Supplementary Figure 5A). It is well known that the expression of PD1 (PDCD1 or CD279) and PD-L1 (CD274) serves as a potential marker for predicting the response to immunotherapy. In our study, we found an important correlation between ABCC1 and CHAC1 expression and the levels of PD1 and PD-L1 (Supplementary Figure 5B). The GSEA analysis of these regulators also targeted meaningful immune-related pathways, such as immune response regulating cell surface receptor signaling, B cell-mediated immunity, and adaptive immune response (Figure 8D). In pan-cancer analysis of 32 cancer species, ABCC1, CHAC1, and GSS may act as hallmarks to activate tumor immune reactions and reshape the tumor environment in LGG, LIGC, BRAC, LUAD, LUSC, and UVM (Figure 8E).

In addition to novel immunotherapies, multiple mRNA inhibitors, including BRAF, also showed an astonishing effect in a large number of patients with sustained efficacy (Luke et al., 2017). Simultaneously, we also identified four mRNAs (AIMP1, TMEM33, ITGB5, and PSPH) with positive polymorphisms in UVM based on the SNP2APA database. The correlation between the three ferroptosis-related modulators and the above mRNA was observed (Supplementary Figure 6).

## Discussion

Uveal melanoma is considered rare but is the most common malignant primary intraocular tumor among adults. Most UVMs are usually treated with surgery or radiotherapy, resulting in similar short-term survival outcomes. With distinct biological and clinical behavior, half of patients will suffer from a poor prognosis, including recurrence and metastatic disease (Marseglia et al., 2021). To date, standard approaches have not provided a survival benefit or precise prognostic prediction for these patients; however, newer systemic therapies, particularly immunotherapies and targeted therapy, have significantly improved patient survival. Furthermore, previous studies have shown that several tumor-infiltrating immune cells, T cells, and dendritic cells are abundant in UVM (de Waard-Siebinga et al., 1996; Polak et al., 2007), indicating that UVM may be a possible target for immunotherapy. However, immune checkpoint inhibitors (ICIs) are remarkably limited by the fact that only a subset of patients with most types of cancer respond to these agents and the unknown mechanisms of innate ICI resistance. In our study, we found that the current TNM classification system is not applicable for predicting prognosis. We performed a retrospective analysis to select biomarkers correlated with the tumor immune microenvironment to predict prognosis and locate that part of the population to benefit most from ICIs.

Ferroptosis is a novel type of cell death that was first proposed by Dixon in 2012 (Dixon, 2017). Many studies have reported that induction of ferroptosis effectively represses tumor development, even in specific drug-resistant tumors (Friedmann et al., 2019; Xu et al., 2019). Notably, the synergistic effect of ferroptosis inducers and PD1/PDL-1 inhibitors resulted in more obvious tumor inhibition. Direct evidence was reported by Wang et al. (2019) that CD8+ T cells have the ability to induce ferroptosis *in vivo* and downregulate the expression of ferroptosis-related genes (SLC7A11). Furthermore, Lang et al. (2019) reported that IFN-γ derived from immunotherapy-activated CD8+ T cells combined with radiotherapy-activated ataxia-telangiectasia mutated (ATM) can strengthen ferroptosis in fibrosarcoma and melanoma cells, which underpins the crosstalk between ferroptosis and anticancer immunity. In contrast, ferroptosis-resistant or ferroptosis-inhibitor-treated tumor cells are insensitive to PDL1 inhibitor treatment (Wang et al., 2019). Thus, based on accumulating evidence, we systematically investigated the expression of 60 ferroptosis-related genes in UVM tumor tissues and their association with OS. Subsequently, we identified 11 regulators by utilizing the Cox analysis in the training set and divided these genes into two modules by MCL clustering in the STRING database. To further verify the biologically plausible hypothesis, UVM cells can communicate with immune cells through a set of ferroptosis-related signals; when clustering by using a consensus clustering analysis according to the expression of genes, the GO, KEGG, and GSEA analyses were implemented to evaluate the function of clusters in two modules; the results revealed that the function of module A was enriched in the primary immunodeficiency, Th1, Th2, and Th17 cell differentiation, which is remarkably related to the immune landscape. Therefore, we confirmed that module A is a key ferroptosis modification in the tumor immune microenvironment. Some researchers have identified the phenomenon through which T cells trigger tumor killing by activating ferroptosis pathways. Nevertheless, the mechanism underlying UVM remains unclear. In the current study, we found that the ESTIMATE score, especially the immune score, was significantly associated with the expression of clusters. Similarly, we validated that the crosstalk of CD8+ T cells and ferroptosis pathways and diverse types of T cells and other TMEs, including B cells, NK cells, macrophages, and dendritic cells, were observed to increase or decrease to have a combined effect on the clinical survival. These results suggest that this module consists of six ferroptosis-related modulators, especially ABCC1, CHAC1, and GSS, which may change the status of TME and exert selective induction of cancer cell death. By focusing on the specific role of the three key ferroptotic genes in TME, results have shown that the level of CD4 memory activated T cells, CD8 T cells, and gamma delta T cells was positively associated with key ferroptotic regulators, which tend to have poor survival. In contrast, naive B cells, resting memory of CD4 T cells, and monocytes were negatively related to these regulators with better survival, suggesting that the sensitivity to ferroptosis is parallel to anticancer immunity.

In our study, we comprehensively explored the predictive value of ferroptosis regulators and constructed two predictive signatures based on the TCGA database. After verifying the validation set, the prognostic model from module A was successfully built; unfortunately, module B failed to be constructed. Compared with the previous clinical characteristics (age, gender, T stage), our prognostic risk signature can achieve an AUC of more than 0.8. Although most GEO databases have limited amounts of samples, this model was re-confirmed, and the overlapping regulators, including ABCC1, CHAC1, and GSS, acted as crucial biomarkers associated with poor OS, PFS, DSS, and PFS.

ABCC1 multidrug resistance protein (1/MRP1) is a member of the C subfamily of ABC transporters that is capable of contributing to chemotherapeutic failure in various cancers by regulating the efflux of chemotherapeutic drugs (Sharom, 2008; Robey et al., 2018). Previous reports have shown that ABCC1 is a negative biomarker associated with a decreased survival rate and an increased risk of relapse in colorectal cancer (Zhao et al., 2020), lung cancer (Fan et al., 2020), and breast cancer (Low et al., 2020). However, the relationship with UVM has not been investigated in detail. Our data showed that the expression of ABCC1 was upregulated in UVM with poor survival, and had a similar trend to the immune score, ESTIMATE score, stromal score, and diverse types of T cells, especially the levels of PD1 and PD-L1, implying that ABCC1 may play a non-negligible role in shaping the TME landscape to affect the therapeutic efficacy of immune checkpoint blockade. CHAC1 is a new pro-apoptotic member of the unfolded protein response pathway and is intimately related to failed chemotherapy (Shuda et al., 2003; Scriven et al., 2009; Goebel et al., 2012). Previously, only Yanchen Liu indicated that the mRNA expression of CHAC1 was associated with poor OS and high risk of metastasis in a limited number of samples from 34 patients (Liu et al., 2019). In the present study, we re-validated the conclusion above in four datasets and further discovered a relationship between CHAC1 and the immune signature of UVM, illustrating the innate mechanisms by which CHAC1 exerts a harmful influence on clinical outcomes. Glutathione synthetase (GSS), which plays a key role in metabolism, catalyzes the last step of glutathione (GSH) synthesis, which is an important antioxidant that protects cancer cells against potential ferroptosis (Proneth and Conrad, 2019; Chen et al., 2020). In liver cancer, researchers found that RRM2 was capable of sustaining intracellular GSH by protecting GSS from degradation (Yang et al., 2020). To the best of our knowledge, this is the first study to explore the correlation between the expression of GSS and the carcinogenic environment of UVM. Interestingly, our findings suggest that the expression of GSS, combined with CHAC1 and ABCC1, mediates the TME infiltration patterns to accelerate UVM progression partly by regulating T cells and other types of immune cells. Tumor burden is considered a promising indicator to promote tumorigenesis in malignant cells and exhibits predictive utility in identifying responders to ICIs. According to the SNP2APA database, we identified positive polymorphisms from four mRNAs and found that all mRNAs were highly correlated with the levels of ABCC1, CHAC1, and GSS, indicating that high expression of key ferroptosis regulators was related to the sharp accumulation of gene mutations, thus becoming a promising therapeutic target and prediction for UVM.

Furthermore, to determine how ferroptosis-related genes act in a lncRNA–miRNA dependent manner in the process of UVM progression, we built a ceRNA network consisting of 25 ferroptosis-related mRNAs, 12 miRNAs, and 29 lncRNAs. In this network, many intersections have been verified in experiments; for example, miR-155 was upregulated, blocking the translation of TP53INP1 to induce cell migration during pancreatic cancer evolution (Seux et al., 2011). Hence, we plan to take the next step to explore the interaction of these molecules *in vitro*.

## Conclusion

This study is the first to comprehensively explore the role of ferroptosis-related genes in UVM and extensively profile the immune landscape based on ferroptosis pathways. Ferroptosis modification clusters, which have different clinical survival, have become an important mediator in the heterogeneity and complexity of the TME. After analyzing the correlation among clusters, we developed and validated a six-gene signature prognostic model to precisely predict the clinical outcome of UVM. Finally, we further evaluated the function of three key prognostic ferroptosis-related regulators, including ABCC1, CHAC1, and GSS, and constructed a ceRNA network to elucidate the molecular mechanisms based on these key regulators. Our findings suggest that ferroptosis-related genes may act as promising indicators for determining effective therapeutic strategies and providing novel insights into immunotherapy.

## Data Availability Statement

The datasets presented in this study can be found in online repositories. The names of the repository/repositories and accession number(s) can be found in the article/Supplementary Material.

## Author Contributions

KC designed the study. YJ and WZW analyzed, interpreted the data, and wrote original draft. WZW, DH, and YZ wrote this manuscript. LG, MX, and XC edited and revised the manuscript. All authors have seen and approved the final version of the manuscript.

## Conflict of Interest

The authors declare that the research was conducted in the absence of any commercial or financial relationships that could be construed as a potential conflict of interest.

## Publisher’s Note

All claims expressed in this article are solely those of the authors and do not necessarily represent those of their affiliated organizations, or those of the publisher, the editors and the reviewers. Any product that may be evaluated in this article, or claim that may be made by its manufacturer, is not guaranteed or endorsed by the publisher.

## Footnotes

1
https://portal.gdc.cancer.gov/

2
http://string-db.org

3
https://sourceforge.net/projects/estimateproject/

4
http://mirtarbase.mbc.nctu.edu.tw/

5
http://www.mirdb.org/

6
http://www.targetscan.org/

7
http://www.R-project.org

8
https://genome-cancer.ucsc.edu

## Supplementary Material

Supplementary Figure 1 Clustering of ferroptosis-related genes in module B. **(A)** Pearson correlation analysis among ferroptosis-related regulators in module B. **(B)** Consensus clustering cumulative distribution function (CDF) and relative change in area under CDF curve for *k* = 2 in module B. **(C)** Kaplan–Meier curves of two clusters in UVM about OS in module B. **(D)** Different expression of the ferroptosis-related mediators in two clusters based on module B (****p* < 0.001; ***p* < 0.01; **p* < 0.05).

Supplementary Figure 2 Tumor immune microenvironment in module B. **(A)** Different expression of ESTIMATE score in two clusters. **(B)** Different distribution of 22 TME infiltrating cells in two patterns (****p* < 0.001; ***p* < 0.01; **p* < 0.05).

Supplementary Figure 3 Prognostic analysis of risk model and ferroptosis-related genes in module B. **(A)** Kaplan–Meier curves of patients in high/low risk about OS in module B. **(B)** Distributions of risk scores, alive/dead status, and expression of ferroptosis-related genes in module B. **(C)** ROC curve of risk score and clinical characteristics in module B. **(D)** Kaplan–Meier curves of patients in high/low risk about OS based on GSE84976.

Supplementary Figure 4 Prognostic value of ferroptosis-related genes. **(A)** Kaplan–Meier DSS curves of patients in key ferroptosis-related genes. **(B)** Kaplan–Meier PFI curves of patients in key ferroptosis-related genes.

Supplementary Figure 5 Evaluation of key ferroptosis-related genes in immune cell infiltration. **(A)** Relationship between ABCC1, CHAC1, and GSS expression and infiltrating immune cells. **(B)** Relationship between key genes expression and level of CD274 and CD279.

Supplementary Figure 6 Correlation of key ferroptosis-related genes and mRNAs with positive polymorphisms. **(A)** Correlation of ABCC1 and mRNAs with positive polymorphisms. **(B)** Correlation of CHAC1 and mRNAs with positive polymorphisms. **(C)** Correlation of GSS and mRNAs with positive polymorphisms.

Supplementary Table 1 Sixty ferroptosis-related genes list.

Supplementary Table 2 Cox analysis of ferroptosis-related genes in TCGA and GSE84976.

Supplementary Table 3 Differential expression lncRNAs based on TCGA.

Supplementary Table 4 Differential expression miRNAs based on TCGA.

Supplementary Table 5 Targeted mRNAs base on miRTarBase, miRDB, and TargetScan databases.

## Abbreviations

UVM: uveal melanoma
SEER: Surveillance, Epidemiology, and End Results
EUROCARE: European Cancer Registry-based study on survival and care of cancer patients
PCD: programmed cell death
RCD: regulated cell death
ROS: reactive oxygen species
DCR: disease control rate
TME: tumor microenvironment
TCGA: The Cancer Genome Atlas
GEO: Gene Expression Omnibus Database
PPI: protein-protein interaction
OS: overall survival
DEGs: differentially expressed genes
GSEA: Gene set enrichment analysis
HR: hazard ratio
ROC: receiver operating characteristic
ceRNA: competing endogenous RNA
PFS: progression-free survival
DSS: disease-specific survival
PFI: progression-free interval
ICIs: immune checkpoint inhibitors.
