# Supplementary figures and images for: Analysis of Ferroptosis-Mediated Modification Patterns and Tumor Immune Microenvironment Characterization in Uveal Melanoma
Source: Front Cell Dev Biol. 2021 Jul 27;9:685120. doi: 10.3389/fcell.2021.685120 (PMC8353259; doi:10.3389/fcell.2021.685120)

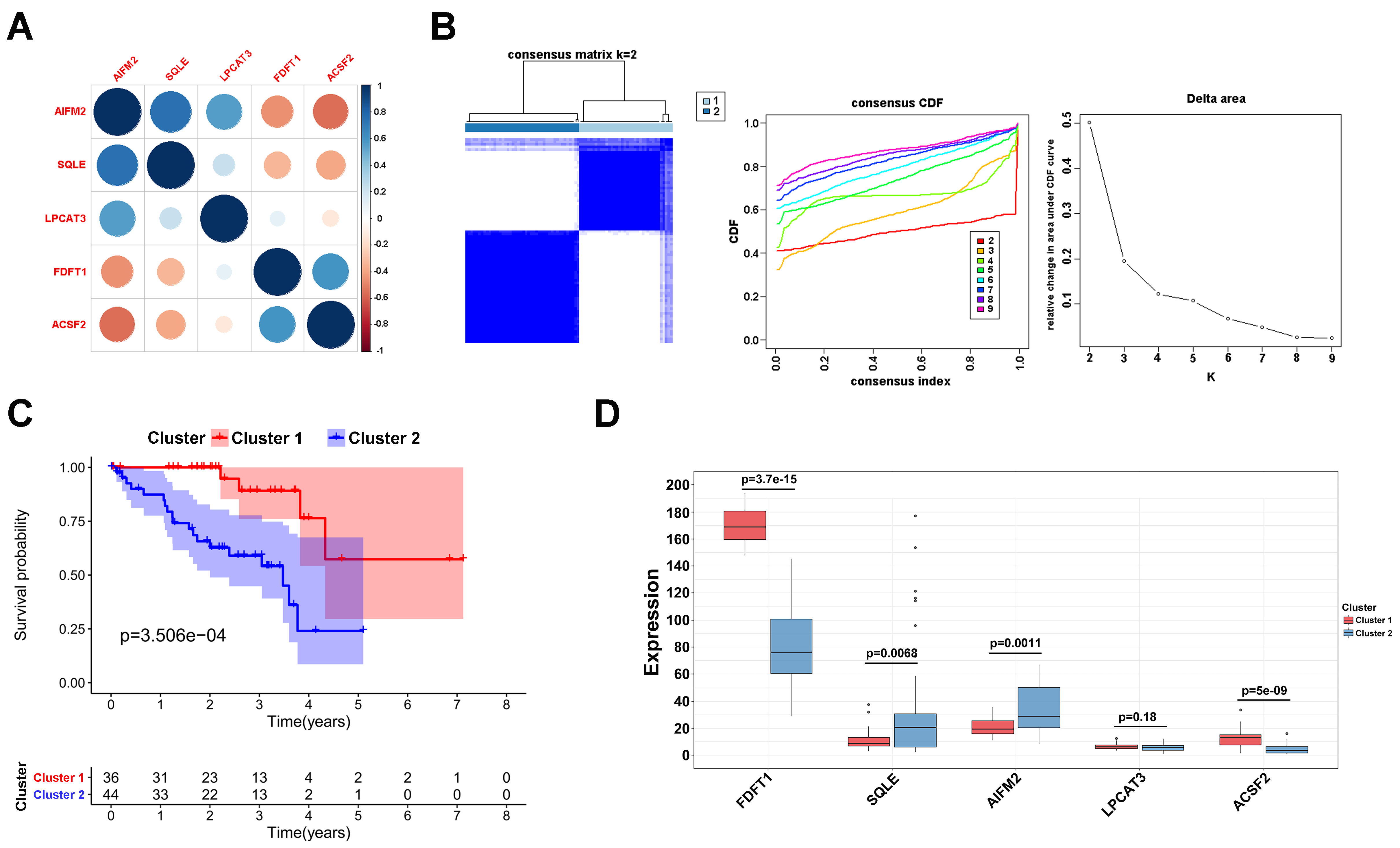

Supplement: Supplementary Figure 1 — Clustering of ferroptosis-related genes in module B. (A) Pearson correlation analysis among ferroptosis-related regulators in module B. (B) Consensus clustering cumulative distribution function (CDF) and relative change in area under CDF curve for k = 2 in module B. (C) Kaplan–Meier curves of two clusters in UVM about OS in module B. (D) Different expression of the ferroptosis-related mediators in two clusters based on module B (***p < 0.001; **p < 0.01; *p < 0.05). [file Image_1.TIF]

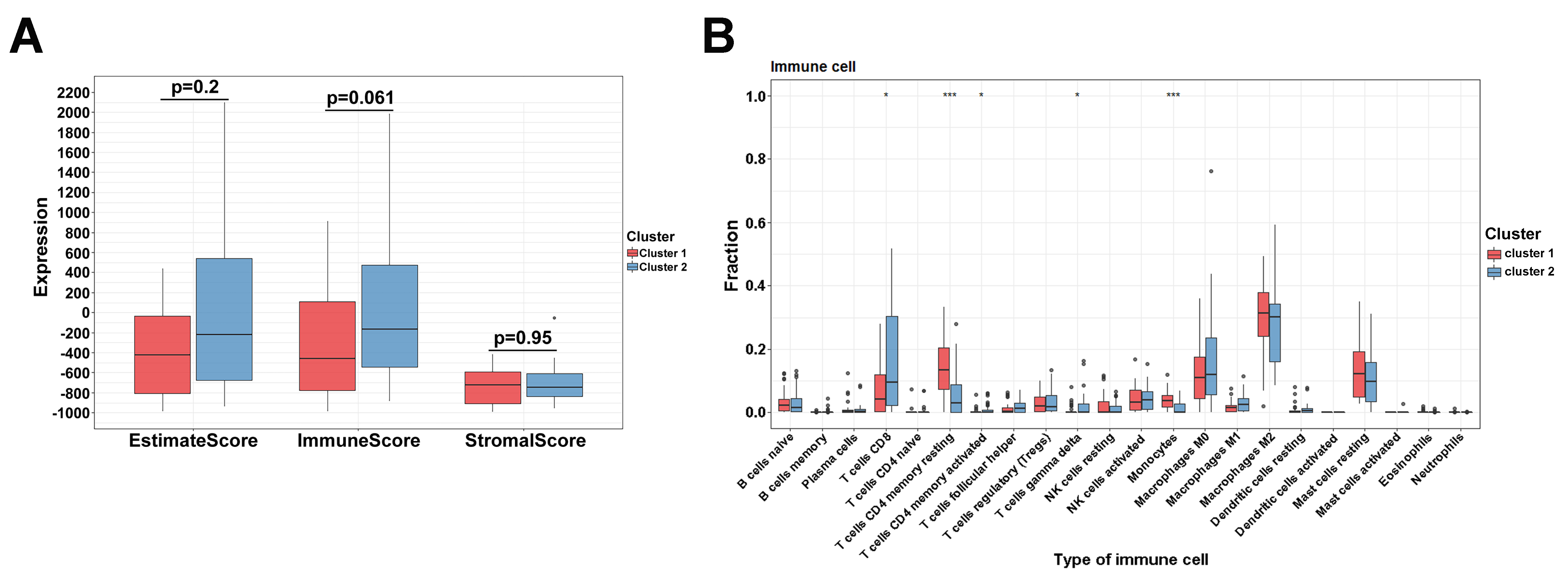

Supplement: Supplementary Figure 2 — Tumor immune microenvironment in module B. (A) Different expression of ESTIMATE score in two clusters. (B) Different distribution of 22 TME infiltrating cells in two patterns (***p < 0.001; **p < 0.01; *p < 0.05). [file Image_2.TIF]

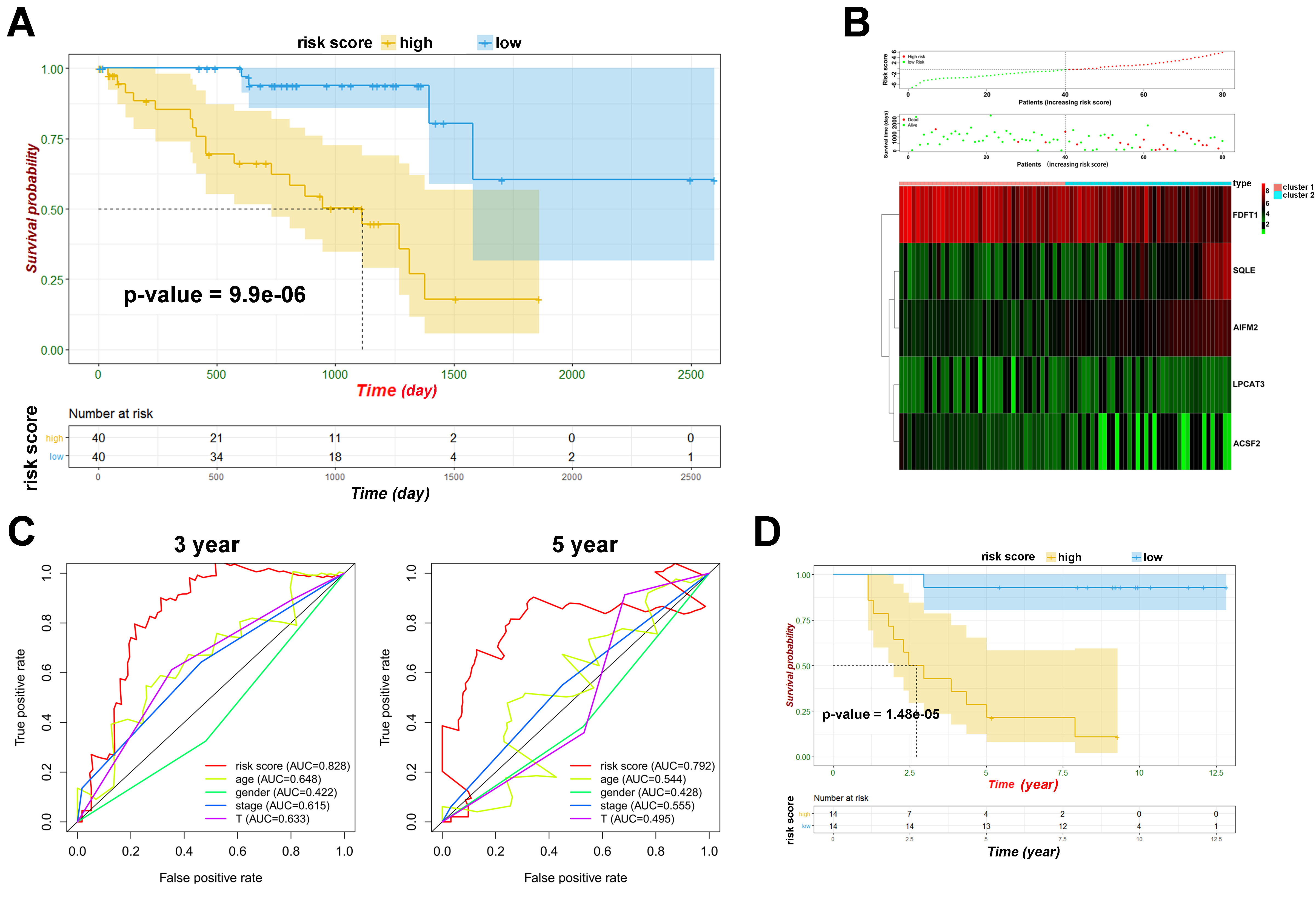

Supplement: Supplementary Figure 3 — Prognostic analysis of risk model and ferroptosis-related genes in module B. (A) Kaplan–Meier curves of patients in high/low risk about OS in module B. (B) Distributions of risk scores, alive/dead status, and expression of ferroptosis-related genes in module B. (C) ROC curve of risk score and clinical characteristics in module B. (D) Kaplan–Meier curves of patients in high/low risk about OS based on GSE84976. [file Image_3.TIF]

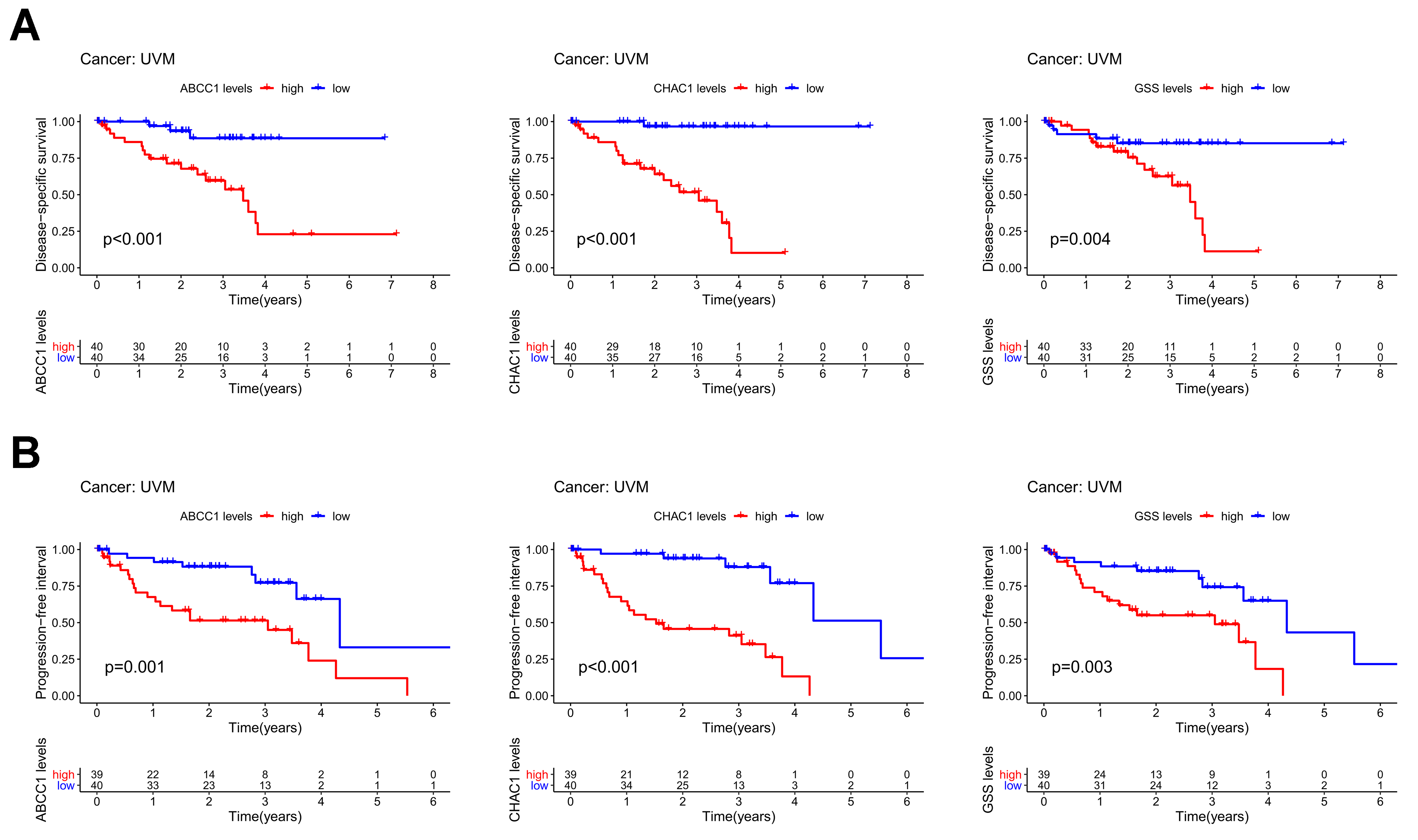

Supplement: Supplementary Figure 4 — Prognostic value of ferroptosis-related genes. (A) Kaplan–Meier DSS curves of patients in key ferroptosis-related genes. (B) Kaplan–Meier PFI curves of patients in key ferroptosis-related genes. [file Image_4.TIF]

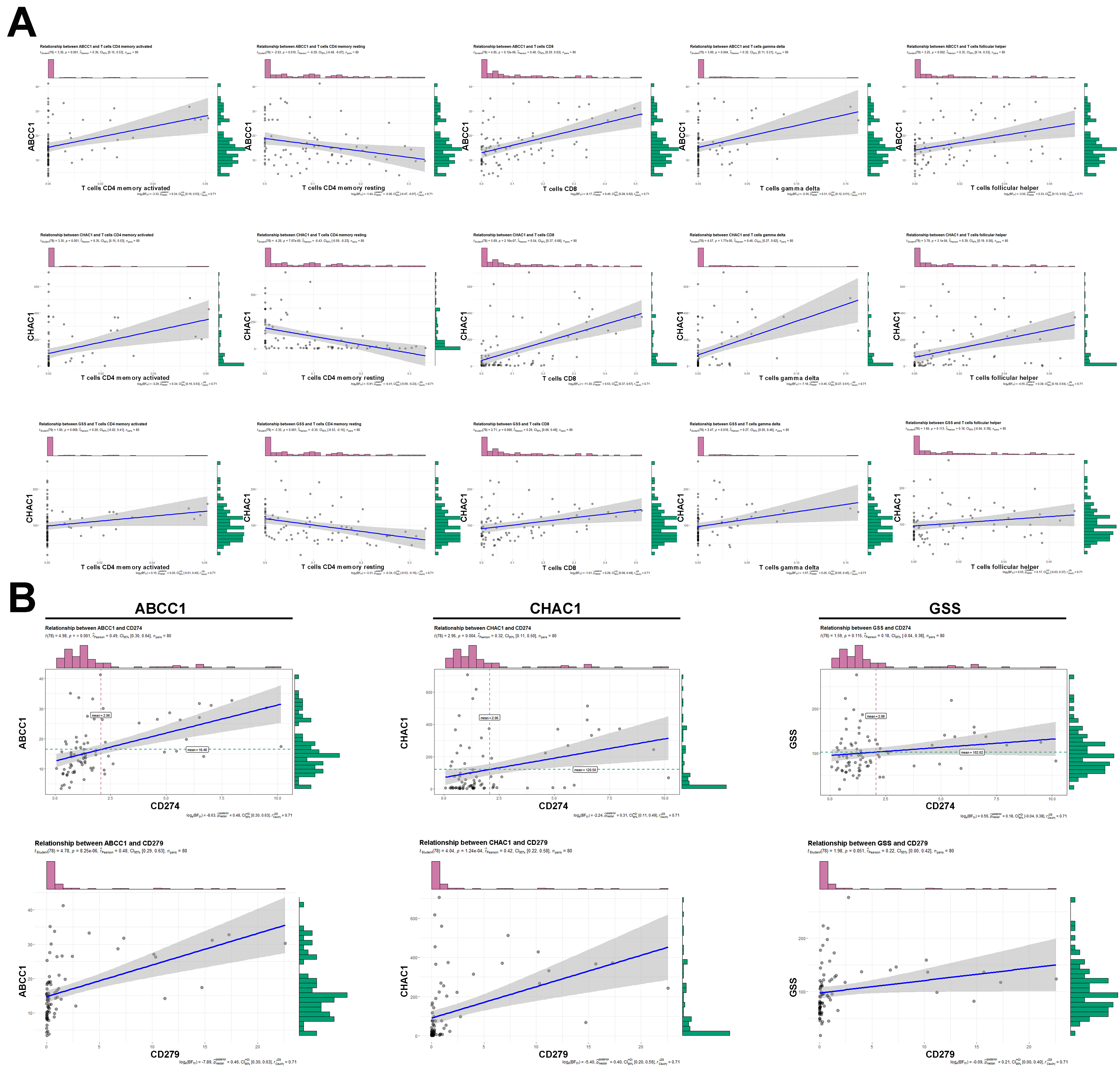

Supplement: Supplementary Figure 5 — Evaluation of key ferroptosis-related genes in immune cell infiltration. (A) Relationship between ABCC1, CHAC1, and GSS expression and infiltrating immune cells. (B) Relationship between key genes expression and level of CD274 and CD279. [file Image_5.TIF]

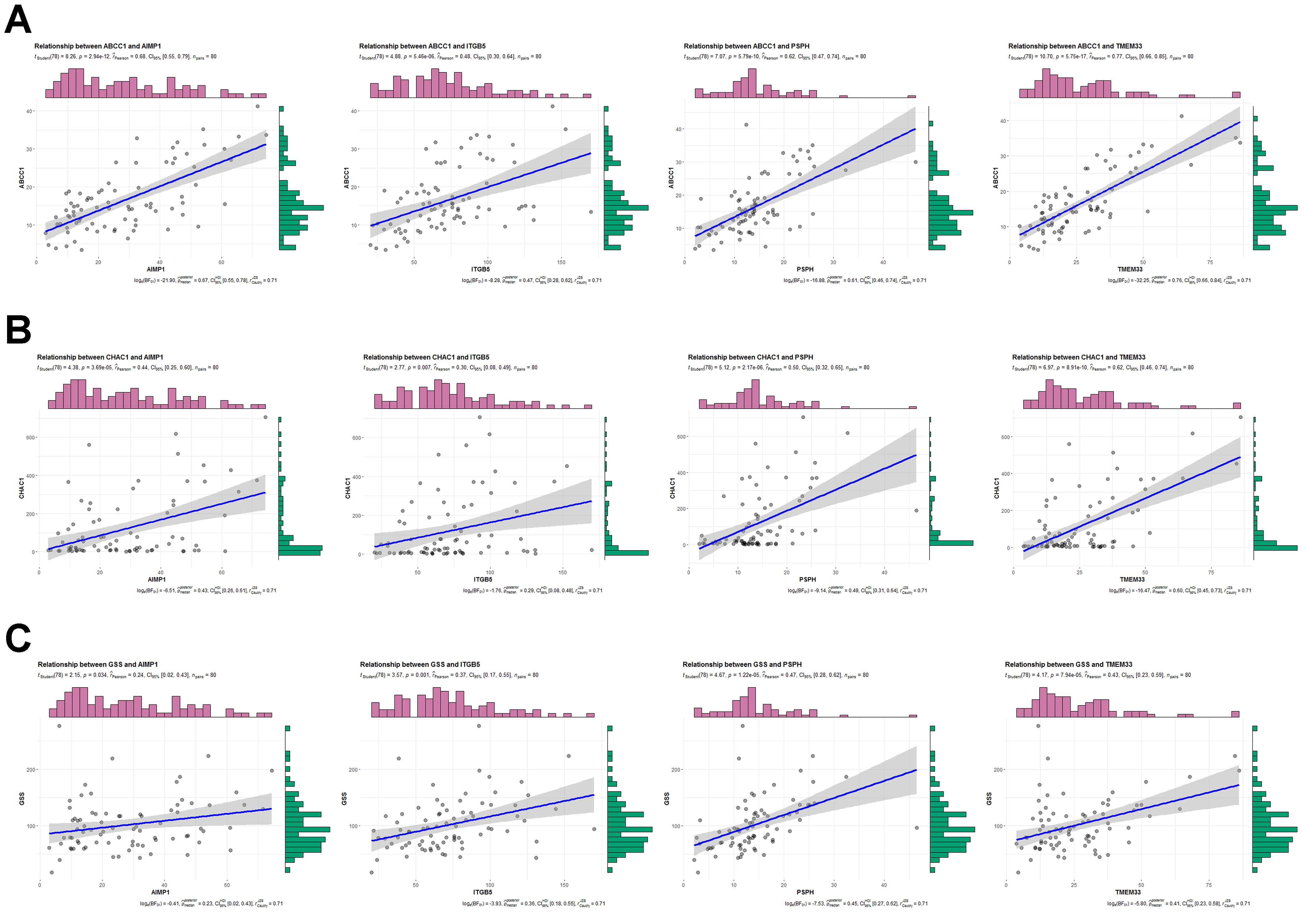

Supplement: Supplementary Figure 6 — Correlation of key ferroptosis-related genes and mRNAs with positive polymorphisms. (A) Correlation of ABCC1 and mRNAs with positive polymorphisms. (B) Correlation of CHAC1 and mRNAs with positive polymorphisms. (C) Correlation of GSS and mRNAs with positive polymorphisms. [file Image_6.TIF]
